# Expanding the Antipsychotic Arsenal: Drug Discovery for Schizophrenia via Virtual Screening and Ligand Optimization of FDA-Approved Drugs

**DOI:** 10.1192/bjo.2026.11951

**Published:** 2026-06-30

**Authors:** Rushank Goyal, Suze van Adrichem

**Affiliations:** Stanford University, Stanford, CA, USA

## Abstract

**Aims:**

Schizophrenia affects 0.43% of global adults, with an estimated annual excess economic burden of $343.2 billion in the United States alone. The schizophrenia drug ecosystem is characterized by significant heterogeneity in patient responses, warranting continual drug discovery efforts to improve outcomes for patients responding suboptimally to existing treatments. Given the centrality of dopamine D2 receptor (DRD2) antagonism in current treatments, this study focused on (i) identifying strong DRD2 binders among Food and Drug Administration (FDA)-approved drugs as repurposing candidates and (ii) generating optimized variants of known antipsychotics as novel drug candidates.

**Methods:**

We developed a computational drug discovery pipeline to score potential drug candidates based on DRD2 receptor binding affinity. Molecular docking scores were calculated by dockstring, a Python wrapper for AutoDock Vina, that automatically finds the best binding pose and returns its binding affinity to the receptor. Virtual screening was performed on:

1. ∼1,600 FDA-approved drugs, with emphasis placed on central nervous system-active drugs known to permeate the blood-brain barrier.

2. 450 variant ligands of six reference antipsychotics (haloperidol, trifluoperazine, fluphenazine, chlorpromazine, thioridazine, and risperidone), generated using SwissSimilarity.

A molecular weight threshold of 500 Da was applied to exclude large ligands, consistent with Lipinski’s Rule of 5 for oral bioavailability. Candidates achieving high binding affinity (≤-9.5 kcal/mol) were further evaluated against existing literature.

**Results:**

Among FDA-approved drugs, risperidone achieved the lowest docking score and therefore the best binding affinity (-11.9 kcal/mol), validating our pipeline by recapitulating a known antipsychotic. Several candidates with high predicted binding affinities (≤-9.5 kcal/mol) were identified, with two of particular interest:

- Dolasetron. Other serotonin 5-HT3 receptor antagonists, especially ondansetron, have improved schizophrenic symptoms in previous studies, making this a promising line of investigation.
- Trazodone. A population-based cohort study published last year offered evidence that trazodone reduced risk of re-hospitalisation for delirium in older patients as compared to other antipsychotics.

Ligand optimization yielded 4 distinct molecules with superior binding affinities relative to their parent compounds. The best-performing variant achieved -13.6 kcal/mol (risperidone-derived), while the largest improvement was observed for thioridazine (-12.7 kcal/mol versus -8.5 kcal/mol original).

**Conclusion:**

An automated DRD2-focused docking workflow can recover known antipsychotics, surface plausible repurposing candidates, and propose optimized variants with improved predicted binding. While these computational hits require further ADMET analysis and experimental validation before strong conclusions are drawn about clinical potential, our results demonstrate the potential utility of computational drug discovery methods in expanding therapeutic options for schizophrenia.

